# Discovery and Characterization of Distinct Simian Pegiviruses in Three Wild African Old World Monkey Species

**DOI:** 10.1371/journal.pone.0098569

**Published:** 2014-06-11

**Authors:** Samuel D. Sibley, Michael Lauck, Adam L. Bailey, David Hyeroba, Alex Tumukunde, Geoffrey Weny, Colin A. Chapman, David H. O’Connor, Tony L. Goldberg, Thomas C. Friedrich

**Affiliations:** 1 Department of Pathobiological Sciences, University of Wisconsin-Madison, Madison, Wisconsin, United States of America; 2 Department of Pathology and Laboratory Medicine, University of Wisconsin-Madison, Madison, Wisconsin, United States of America; 3 Makerere University, Kampala, Uganda; 4 Department of Anthropology and McGill School of Environment, Montreal, Quebec, Canada; 5 Wisconsin National Primate Research Center, Madison, Wisconsin, United States of America; University of California Riverside, United States of America

## Abstract

Within the *Flaviviridae*, the recently designated genus *Pegivirus* has expanded greatly due to new discoveries in bats, horses, and rodents. Here we report the discovery and characterization of three simian pegiviruses (SPgV) that resemble human pegivirus (HPgV) and infect red colobus monkeys (*Procolobus tephrosceles*), red-tailed guenons (*Cercopithecus ascanius*) and an olive baboon (*Papio anubis*). We have designated these viruses SPgV_krc_, SPgV_krtg_ and SPgV_kbab_, reflecting their host species’ common names, which include reference to their location of origin in Kibale National Park, Uganda. SPgV_krc_ and SPgV_krtg_ were detected in 47% (28/60) of red colobus and 42% (5/12) red-tailed guenons, respectively, while SPgV_kbab_ infection was observed in 1 of 23 olive baboons tested. Infections were not associated with any apparent disease, despite the generally high viral loads observed for each variant. These viruses were monophyletic and equally divergent from HPgV and pegiviruses previously identified in chimpanzees (SPgV_cpz_). Overall, the high degree of conservation of genetic features among the novel SPgVs, HPgV and SPgV_cpz_ suggests conservation of function among these closely related viruses. Our study describes the first primate pegiviruses detected in Old World monkeys, expanding the known genetic diversity and host range of pegiviruses and providing insight into the natural history of this genus.

## Introduction

Pegiviruses are single-stranded, positive-sense RNA viruses within the family *Flaviviridae*. This family includes three additional genera, *Hepacivirus*, *Pestivirus*, and *Flavivirus*, and encompasses a diverse set of viruses, including important human pathogens such as hepatitis C virus (HCV), a hepacivirus first described in 1989 [1]. The discovery in 1995 of human pegivirus (HPgV) [2], formerly known as GB virus C or hepatitis G virus [3], [4], was the product of efforts to uncover hepatitis viruses related to HCV. While HPgV is the human virus most closely related to HCV, it does not cause hepatitis [5]. Other early work to discover HCV-like viruses focused on nonhuman primates and revealed pegiviruses distantly related to HPgV in sera from six New World monkey species [6], [7] and variants closely related to HPgV in sera from captive and wild chimpanzees [2], [8], [9]. After 1998, few new pegiviruses were reported until 2013, a year that witnessed a flourishing of pegivirus discoveries in horses [10], [11], bats [12], [13], and rodents [14], [15]. These recent revelations suggest that pegivirus infection may be widespread among mammals, although, until now, no related viruses had been identified in Old World monkeys.

Among the pegiviruses, HPgV has garnered the greatest attention. HPgV establishes an asymptomatic infection and circulates as at least five closely related, yet phylogenetically distinct, genotypes that cluster geographically [16]. HPgV is thought to be predominately lymphotropic, and the virus is produced *in vitro* by T- and B-lymphocytes obtained from infected individuals (reviewed in [3]). HPgV infection may persist for decades, although the majority of infections are cleared within two years in healthy individuals [17]. Unlike HCV infection, HPgV viremia is not usually coincident with detectable antibodies against envelope glycoprotein protein 2 (E2), suggesting that HPgV infection is resolved efficiently once a humoral immune response to E2 is mounted [18]. Based on detection of the virus in blood donors, the prevalence of HPgV infection is significantly lower in developed (1–5%) versus developing (∼20%) countries [19]. Higher HPgV prevalence (∼20–40%) is associated with sexual and parenteral risk behaviors and, accordingly, with human immunodeficiency virus type 1 (HIV) and HCV infections [20]–[23]. Although the cellular tropism of pegiviruses infecting animals is largely undetermined [3], the characteristics of pegivirus infection in nonhuman hosts appear to closely resemble those observed in humans: virus can be detected at high titer in blood, infection may persist for some time, and a remarkably low degree of within-host viral genetic diversity is observed in time-series samples [7], [19], [24]. Interestingly, Theiler’s disease, an infectious hepatitis in horses, is the only disease associated with a pegivirus infection [10].

In the past decade, research on HPgV has developed from understanding the virus’s evolution and phylogeography [24]–[26] to clarifying its medical importance in relation to HIV infection. In HIV-positive individuals, HPgV viremia has been associated with prolonged survival and a milder HIV disease course, including higher CD4+ T cell counts, lower HIV viral load, and delayed progression to AIDS (reviewed in [18]). Impacts of HPgV infection that potentially contribute to this antagonism include direct antiviral effects; altered expression of cytokines, chemokines and HIV entry receptors; and modulation of host cell signaling pathways [18], [19], [27]. However, the mechanisms underlying these phenomena are still not fully understood, and no tractable animal models exist to study them.

Here we report the discovery and characterization of three pegiviruses infecting red colobus monkeys (*Procolobus tephrosceles*), red-tailed guenons (*Cercopithecus ascanius*) and an olive baboon (*Papio anubis*) from Kibale National Park, Uganda. After simian pegiviruses (SPgV) identified in chimpanzees (SPgV_cpz_), these viruses are the closest known relatives to HPgV. Their discovery in Old World monkeys expands the known host range of the pegiviruses and provides new insights on their natural history and evolution.

## Results

### Discovery of Novel Simian Pegiviruses

This study was conducted as part of a long-term investigation of health and conservation focused on the region of Kibale National park, western Uganda [28]. Kibale is noted for its high diversity and density of non-human primates, which host a diversity of pathogens [29]–[34], and which interact extensively and often antagonistically with local people and domestic animals [28]. Deep sequencing of plasma RNA from wild red colobus monkeys (*Procolobus tephrosceles*), red-tailed guenons (*Cercopithecus ascanius*) and olive baboons (*Papio anubis*) revealed the presence of three distinct, host-associated viruses with greatest similarity (via BLAST) to human and chimpanzee pegiviruses. *De novo* assembly and iterative mapping of sequencing reads recovered near-complete genomes comprising a single continuous open reading frame (ORF) and partial 5′- and 3′-untranslated regions (UTR) from most positive animals (GenBank sequence accession no. KF234499 to KF234530). According to recently adopted nomenclature [3], we tentatively designated the new viruses SPgV_krc_, SPgV_krtg_ and SPgV_kbab_ to indicate their host species of origin (Kibale red colobus, Kibale red-tailed guenon and Kibale olive baboons, respectively) and their shared common ancestry with members of the *Pegivirus* genus. Sequencing statistics, detection frequencies and viral titers in plasma, measured by TaqMan qRT-PCR, are summarized in Table 1. SPgV_krc_ and SPgV_krtg_ were detected in 47% (28/60) of red colobus and 42% (5/12) red-tailed guenons, respectively, while SPgV_kbab_ infection was detected in just one baboon (of 23). High viral titers were documented, consistent with observations made for PgV infections in other mammals (Fig. S1) [10], [12], [35]. No animals appeared clinically ill at the time of sampling, nor have any been subsequently observed with overt clinical signs. Attempts to isolate these viruses are ongoing.

**Table 1:** Detection frequency, TaqMan qRT-PCR titer and genome statistics for Kibale SPgVs.

	ORF (nt)	Detection Frequency	Sequenced Reads^a^	Ave. Coverage Depth^a^	Sequence Length (nt)^a^	Titer (g.c. mL^−1^)^a^
SPgV_kbab_	8652	1/23	29023	402	9434	2.4×10^7^
SPgV_krc_	8697	28/60	548–312763	5–6264	8776–9596	1.8×10^4^–2.2×10^8^
SPgV_krtg_	8631	5/12	1321–6630	21–101	9119–9284	2.1×10^6^–2.1×10^7^

a Ranges for positive animals; assays were sensitive to between 10 and 100 genome copies (g.c.) per 20-µL qRT-PCR reaction.

### Genome Characterization of SPgV_krc_, SPgV_krtg_ and SPgV_kbab_

Like all *Flaviviridae*, the genomes of the newly discovered Kibale SPgVs encode a single, continuous ORF (8552 to 8697 nucleotides, nt). Cleavage site prediction along the polyprotein distinguished nine mature proteins representing the typical proposed *Pegivirus* genome organization: E1/E2/p7/NS2/NS3/NS4A/NS4B/NS5A/NS5B (see below). The translation initiation codon is poorly defined for several pegiviruses [3], [9], [13]. For each of the Kibale SPgVs, the first Met in-frame with the coding sequence aligned well with the experimentally determined translation initiation codon for HPgV [36]. Measured from this position, the aligned coding sequence lengths for the Kibale SPgVs were similar to those reported for SPgV_cpz_ (8484 nt) and HPgV (8529 to 8565 nt) but were considerably shorter than those of the New World simian pegiviruses (8865–9018 nt) and the divergent pegiviruses recently reported in bats (BPgV; 8919–9777 nt), rodents (RPgV; 9855 nt) and horses (EPgV; 9570–9918).

Partial 5′UTR sequences of 498, 513 and 521 nt were recovered by deep sequencing for SPgV_krtg_, SPgV_kbab_, and SPgV_krc_, respectively. These sequences demonstrated significant identity to the 554-nt HPgV 5′UTR. Accordingly, structures analogous to stem-loops II, IIIa, IIIb, IVa, IVb, Va and Vb in the HPgV 5′UTR internal ribosome entry site [36], [37] could be confidently resolved via RNAfold and RNAalifold analyses [38], [39] (Fig. S2). Partial 3′UTR sequences of 147, 162 and 308 nt were recovered for SPgV_krtg_, SPgV_kbab_, and SPgV_krc_, respectively. Compared with the 312-nt HPgV 3′UTR, conservation between the Kibale SPgVs, HPgV and SPgV_cpz_ was strong within the first ∼110 nt. In particular, a pyrimidine-rich poly(C) region, reminiscent of motifs potentially involved with RNA replication and/or stabilization observed in the 3′UTRs of HCV [40]–[42] and of several mammalian mRNAs [43], was well conserved among the Old World primate pegiviruses. Surrounding the poly(C) region, 3′UTR structures analogous to proposed structural motifs V through VII (as defined in [44]) were apparent, with the poly(C) region always beginning in the loop of structure VI (Fig. S2). Attempts to amplify full 5′- and 3′UTR sequences via RACE and 5′-3′ ligation [45] were unsuccessful.

**Figure 1:**
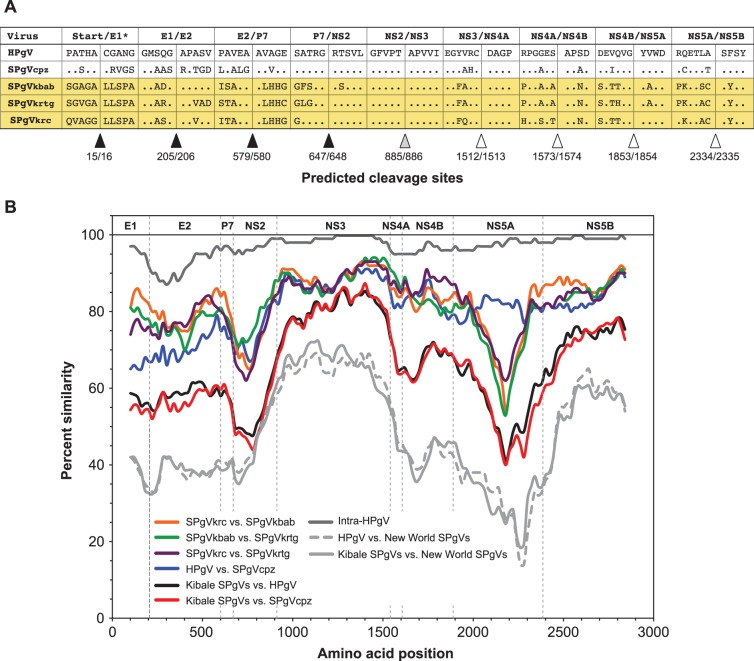
Polyprotein cleavage sites and amino acid similarity of the Kibale SPgVs and related primate pegiviruses. (A) Amino acid sequences of the Kibale SPgVs and related viruses adjacent to predicted protease cleavage sites. Proposed cleavage sites for signalase (black triangles), NS2–NS3 protease (gray triangle), and NS3-4A protease (white triangles) are indicated. Amino acid positions of cleavage sites in relation to SPgV_krc_ are included below the triangles. *The signalase cleavage site predicted for the Kibale SPgVs between Start and E1 (amino acid position 15/16) is located 5′ of those predicted for HPgV and SPgV_cpz_ (amino acid positions 22/23 and 21/22, respectively) in the sequence alignment; all other cleavage sites are aligned among these viruses. (B) Sliding window amino acid similarity among primate *Pegiviruses* across aligned coding regions (window, 200 aa; step, 20 aa). Dashed vertical lines indicate start positions of inferred viral proteins. The dark gray trace demonstrating the high similarity among known HPgV variants was included for reference.

Mature proteins encoded by the 5′ half of *Flaviviridae* genomes are cleaved from the polyprotein by host signal peptidase and were assigned for the Kibale SPgVs through manual [46] and *in silico* [47], [48] signalase cleavage site prediction. Proposed signal-sequence cleavage sites were well conserved among the Kibale SPgVs, HPgV and SPgV_cpz_ (Fig. 1A). Accordingly, two envelope proteins (E1 and E2) and a p7 protein (∼7.5 kDa; ExPASy server [49]) were predicted for these new SPgVs in this region. As observed for other pegiviruses, the Kibale SPgVs apparently lack sequence encoding a core (i.e., nucleocapsid) protein [3], [9], and no alternate reading frame proteins were detected. No major insertions or deletions were observed in structural proteins for the new SPgVs relative to HPgV and SPgV_cpz_. This differs from New World SPgVs, BPgVs, RPgVs and EPgVs, viruses with large insertions (107–183 amino acids, aa) near the C-terminus of E2 and for which signalase cleavage sites in this region are unresolved [13], [14], [50]. It has been speculated that the inserts in these viruses may, in fact, constitute an additional structural protein, designated “X” [13], [14].

Reduced glycosylation of envelope proteins is a notable feature distinguishing HPgV from the hepaciviruses HCV and GB virus B, whose envelopes are heavily glycosylated [3], [19]. Within E1, a single potential glycosylation site (N_135_ with reference to, *wrt*, the SPgV_krc_ ORF) was predicted for the Kibale SPgVs. This site was conserved in roughly the same location among all identified pegiviruses, and no pegivirus examined had more than two predicted potential E1 glycosylation sites. Across E2, a single potential glycosylation site was conserved among nearly all pegiviruses (N_241_ *wrt* SPgV_krc_), and a second site was conserved among all Old World primate pegiviruses (N_400_ *wrt* SPgV_krc_). In total, the Kibale SPgVs each exhibited three potential E2 glycosylation sites, similar to HPgV (three to four), SPgV_cpz_ (three) and New World SPgVs (three to four). Excluding the putative “X” protein region from this analysis, the remaining pegiviruses show similar predicted potential E2 glycosylation (one to four sites) with the exception of the phylogenetically basal clade comprising BPgVs and RPgV (see below), which exhibit six to eight predicted sites.

Non-structural proteins for the members of the family *Flaviviridae* are cleaved by viral nonstructural proteins 2 (NS2) and 3 (NS3), along with NS3’s cofactor NS4A [51], [52], and were identified based on alignment with identified cleavage sites for other pegiviruses [53], [54] and hepaciviruses [51]. The carboxy termini of NS3 and NS4A were approximated due to the current absence of experimental data for related pegiviruses [55]. Amino acids necessary for NS2-mediated autocatalytic cleavage of NS2/NS3 (His_809_, Glu_829_ and Cys_850_, *wrt* SPgV_krc_) and NS3-4A-mediated cleavage of the remaining non-structural proteins (His_941_, Asp_965_ and Ser_1023_, *wrt* SPgV_krc_) were conserved among the Kibale SPgVs. In addition, residues within the NS3 helicase [56] and NS5B RNA-dependent RNA polymerase (RdRp) motifs [57] were highly conserved (91% and 90% amino acid identity, respectively) among the Kibale SPgVs.

Within NS5A, the zinc-binding motif, Cx^17^CxCx^22^C, which is conserved in nearly the same form among hepaciviruses and other related viruses and plays a role RNA replication [58], was conserved among all the pegiviruses examined. The NS5A protein of HCV is known to have intrinsically disordered regions (IDRs) that are important for many of its inferred functions, including modulating host regulatory and signaling processes [59], [60]; the recently discovered guereza hepacivirus (GHV) has a similar IDR [61]. IDRs lack a well-defined three-dimensional structure under native conditions [62] but may undergo substrate-induced disorder-to-order transitions allowing for their interactions with multiple binding partners [63]. To determine if these features were conserved across the hepaci- and pegivirus genera, we searched for IDRs within the sequences of all Old World primate pegiviruses. IDRs were identified in the C-terminal half of NS5A for the Kibale SPgVs (residues ∼2050 to ∼2210). In this ∼160-aa region, 44% (SPgV_krtg_), 67% (SPgV_krc_) and 78% (SPgV_kbab_) of the amino acids were predicted to be intrinsically disordered [62]. The same analysis also predicted disorder in the 3′ end of NS5A for HPgV (73% of residues 2065 to 2175, *wrt* NC_001710) and SPgVcpz (82% of residues 2053 to 2154; *wrt* AF070476). Using ANCHOR [64], IDRs with tendency to undergo substrate-induced disorder-to-order transitions were detected in each of the viruses examined, suggesting that the pegivirus IDRs may have the capacity to bind transiently to a range of substrates. In particular, major TNFR-associated factor 2 (TRAF2) binding motifs were detected within the IDRs of each of the Old World primate pegiviruses (*p* = 0.0043, [65]). The consistent identification of TRAF2 binding motifs in IDRs suggests a potential role for NS5A in modulating signal transduction from members of the tumor necrosis factor (TNF) receptor family [66], [67].

### Similarity Analysis

Pairwise comparison of complete open reading frame (ORF) sequences demonstrated that the three Kibale SPgVs shared ∼73% nucleotide (nt) and ∼81% amino acid (aa) identity (ID) with each other but were approximately equally divergent from HPgV (63% nt ID; 65% aa ID), SPgV_cpz_ (62% nt ID; 65% aa ID), New World SPgVs (53% nt ID; 48% aa ID), and more-divergent pegiviruses infecting bats, horses and rodents (45–54% nt ID; 34–49% aa ID). ORF sequences for Kibale SPgVs infecting the same monkey species were highly similar (≥96% nt ID).

Sliding-window similarity analysis across complete ORF sequences illustrated the degree of amino acid identity of the Kibale SPgVs to closely related pegiviruses (Fig. 1B). The Kibale SPgVs were essentially equally divergent from one another (Fig. 1B, orange, green and purple traces), averaging 78% ID across E1 and E2 and 89% ID within NS3. Interestingly, no Kibale SPgV pair was consistently most similar across the ORF. Together, these viruses shared greatest sequence identity with HPgV and SPgV_cpz_ variants (Fig. 1B, black and red traces, respectively), averaging 57% ID across E1 and E2 and 81% ID in NS3. Nearly equal divergence was observed between groups comprising the New World SPgVs and the Kibale SPgVs (Fig. 1B, solid light gray trace) or the New World SPgVs and the pegiviruses detected in humans and chimpanzees (HPgV+SPgV_cpz_ group) (Fig. 1B, dashed light gray trace). Notably, this comparison excludes variability in the C-terminus of E2, where unique insertions are present in New World SPgVs (and other divergent pegiviruses); identity of these viruses with Old World primate pegiviruses is therefore undefined in this genomic region. The greatest similarity among all the pegiviruses compared was observed over conserved NS3 helicase and NS5B RdRp motifs (17, 18), corresponding to the essential roles the encoded proteins play in the *Flaviviridae* replication cycle. Minima in sequence identity were observed in NS2 and, in particular, in the C-terminal half of NS5A, where considerable variability in sequence length and identity exists among many of the pegiviruses. This region is also where an unusual twelve-amino-acid insert was noted for the so-called “indel type” HPgVs [68]. Interestingly, the characteristic decline in NS5A sequence identity observed here and elsewhere between different pegiviruses [14] was absent between HPgV and SPgV_cpz_ (Fig. 1B, blue trace), perhaps reflecting the close relationship between humans and chimpanzees.

### Phylogenetic Analyses

**Figure 2:**
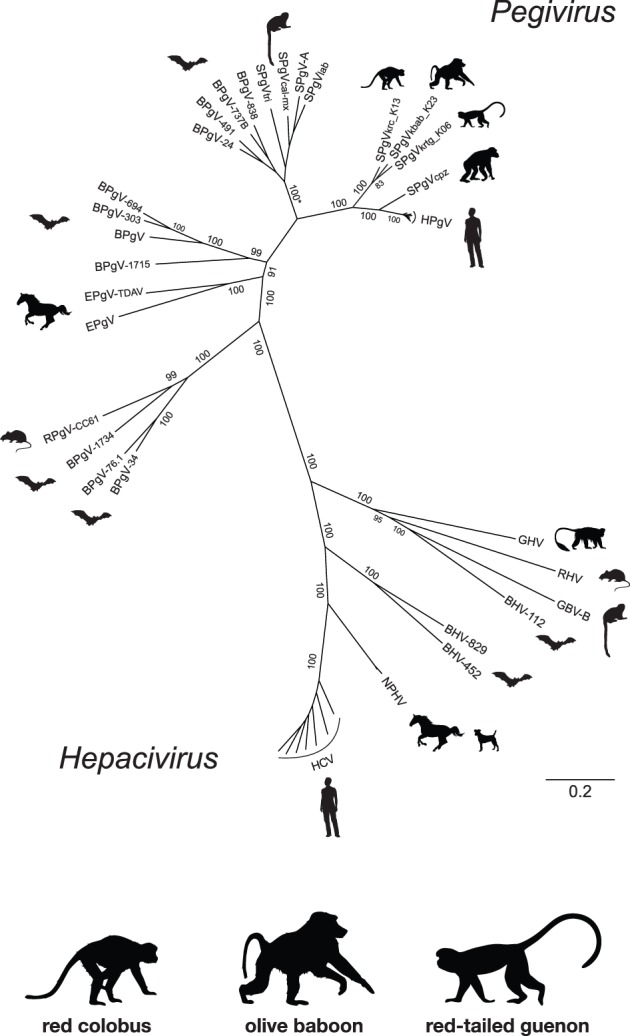
Bayesian phylogeny of the Kibale simian pegiviruses, SPgV_krc_, SPgV_krtg_ and SPgV_kbab_, and representative members of the *Pegivirus* and *Hepacivirus* genera. Codon-guided sequence alignments of open reading frame protein sequences were generated via MAAFT, cleaned using Gblocks, and stripped of the third codon position, resulting in a 42-taxa, 3668-character alignment. Viruses representing the known diversity within each clade were chosen for inclusion in the Bayesian analysis. Posterior clade probabilities are shown for major branches. Scale bar indicates nucleotide substitutions per site. *Posterior probabilities for all branches in this clade are 100. GenBank accession numbers for the included taxa are provided in Table S1

To estimate evolutionary relationships of the novel Kibale SPgVs to related viruses, we constructed a Bayesian phylogenetic tree comprising 44 ORF sequences, representing the full available genetic diversity within each major hepaci- and pegivirus clade (Fig. 2). This phylogeny yielded topologies consistent with established relationships among the hepaci- and pegiviruses [3] and demonstrates the relationships among several recently described viruses infecting horses, bats and rodents [10]–[12], [14]. The new Kibale SPgVs are monophyletic and share a most recent common ancestor with human and chimpanzee pegiviruses. Interestingly, the evolutionary distance between the Kibale SPgVs and HPgV is considerably less than the distance observed between HCV and its closest known relative, nonprimate hepacivirus (NPHV), whose natural host is horses [69], [70].

Based on our Bayesian analysis, the phylogenetic relationship among the Old World primate pegiviruses is consistent with that of their primate hosts [71], suggesting the possibility of virus-host co-speciation. However, we note that the relationship among the Kibale SPgVs varied when we used nucleotide versus amino acid alignments for phylogenetic reconstruction (not shown), an observation reflected by the relatively low posterior support obtained for the internal node of the Kibale SPgV clade (83%; Fig. 2). The relationship among the three viruses observed in the similarity plot (Fig. 1B) supports an effective polytomy among the Kibale SPgVs, in that no pair of the three Kibale SPgVs was consistently most similar across the polyprotein.

**Figure 3:**
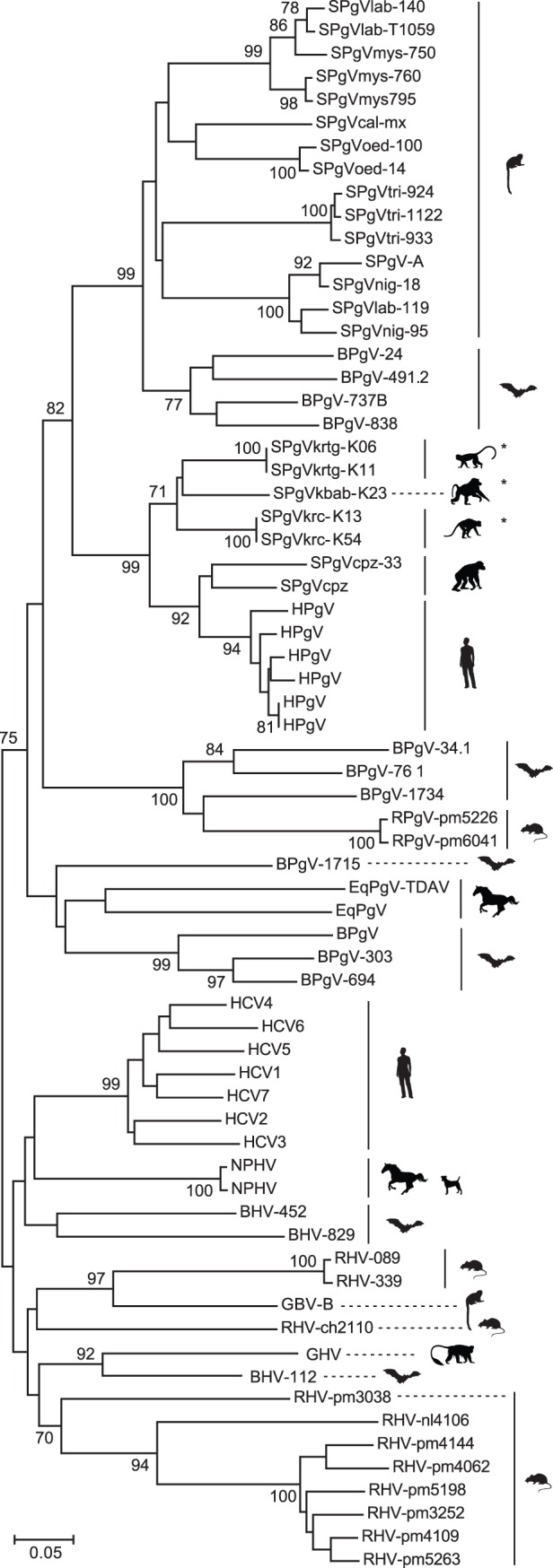
Neighbor-joining amino acid phylogeny of the NS3 helicase comprising 43 pegivirus and 25 hepacivirus sequences. This 97-aa segment (polyprotein positions 1221 to 1317 relative to HPgV, NC_001710) is highly conserved among the *Flaviviridae* and has been targeted extensively for virus discovery and phylogenetic characterization. The sequences included in this analysis encompass the full genetic diversity of identified pegiviruses within each clade, minus those for which NS3 sequence data were unavailable, namely pegiviruses infecting the common marmoset, *Callithrix jacchus* [7] and several recently identified viruses infecting bats [12]. Inclusion of the two most diverse variants of both SPgV_krc_ and SPgV_krtg_ demonstrated the relatively high within-host similarity of these viruses within the study population. GenBank accession numbers for the included taxa are provided in Tables S1 and S2.

A comprehensive neighbor-joining phylogeny based on a highly conserved, 97-aa region of the NS3 helicase gene (Fig. 3), for which abundant sequence information is available for primate pegiviruses and phylogenetic comparisons are often reported, yielded a similar topology to the Bayesian tree [7]–[9], [72], [73]. Sequences included in this phylogeny encompass the full genetic diversity of identified pegiviruses within each clade, with the exception of pegiviruses infecting common marmosets (*Callithrix jacchus*) [7] and several recently identified viruses infecting bats [12], for which NS3 sequences were unavailable. As documented previously [8], chimpanzee pegivirus sequences were monophyletic and considerably more divergent than the most divergent HPgVs. Additionally, these SPgV_cpz_ variants alone exhibited diversity rivaling that observed among the three Kibale SPgVs. Overall, with the addition of the Kibale SPgVs, the between-host genetic diversity of Old World primate pegiviruses now approaches that observed for pegiviruses infecting New World primates. A striking feature of both phylogenies is the paraphyletic (i.e., multi-lineage) distribution of pegiviruses and hepaciviruses infecting bats and rodents, which confirms that these hosts are significant reservoirs for hepaci- and pegivirus genetic diversity [74].

### Within-host Genetic Diversity

We characterized the within-host genetic diversity of each simian pegivirus detected in Kibale animals by quantifying and mapping the distributions of single nucleotide polymorphisms (SNPs) detected in deep sequencing reads along the genomic ORF. Among all variants, within-host genetic variability was low and accounted for at most 100 SNPs using a 5% SNP frequency cutoff. This level of diversity is similar to observations made for HPgV [75] and guereza hepacivirus [61], which infects black-and-white colobus monkeys in Kibale, but is considerably lower than SNP variation observed for HCV [76].

**Figure 4:**
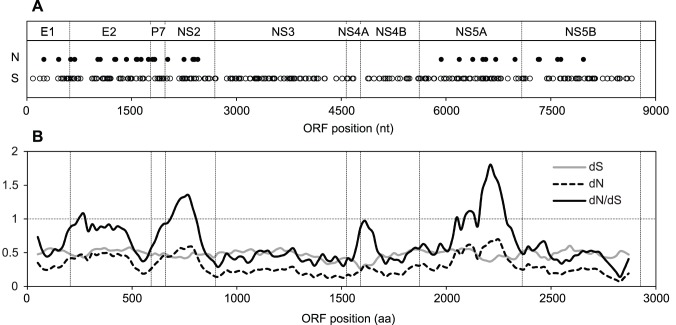
Within- and between-host patterns of selection. (A) Mapping of the distributions of synonymous (S) and nonsynonymous (N) single nucleotide polymorphisms along the ORF for 13 of 28 red colobus samples, for which coverage-depth was greater than 100 reads across most (≥96%) of the ORF. (B) Distribution of average substitution rates at non-synonymous (dN) and synonymous (dS) sites, and their ratio (dN/dS), along a sliding window (100 aa window, 20 aa step) for the comparison of selection pressures among the Kibale SPgVs (accession no. KF234523, KF234526 and KF234530).

Among the SPgVs identified in red colobus, 13 of 28 samples met our variant-calling criteria for coverage depth (≥100 sequences) across most of the ORF (≥95%), exhibiting average coverage depths of 577 to 6264 reads. For these samples, we expect the distribution of variants detected to approximate the true within-host genetic diversity since the entire ORF was eligible for variant calling; for this reason we focused our analysis of within-host genetic diversity on this subset of SPgV_krc_ sequences. Synonymous and nonsynonymous substitutions in this subset of samples varied from 0 to 91 (median = 15) and 0 to 9 (median = 3), respectively. To determine whether the spatial distribution of synonymous and nonsynonymous substitutions along the polyprotein was homogeneous for SPgV_krc_, we aggregated SNPs from these 13 samples, mapped them along the ORF and quantified the percent of total SNPs falling within each gene (Fig. 4A; Table S3). Overall, we observed a total of 318 synonymous and 55 nonsynonymous substitutions in this cohort. The distribution of synonymous substitutions was proportional to the gene length (R^2^ = 0.97; Fig. S3), and approximately 50% occurred at frequencies less than 10%. This pattern suggests a steady and essentially homogeneous contribution of random mutations to within-host synonymous genetic diversity. Conversely, the distribution of nonsynonymous substitutions showed poor correlation to the gene length across the ORF (R^2^ = 0.15; Fig. S4). A clear bias existed towards nonsynonymous substitution within E2, P7, NS2, NS5A and NS5B, and nonsynonymous substitutions were absent in NS3, NS4A and NS4B.

Coverage depths for the five SPgV_krtg_ samples (averaging 21 to 101 reads) were lower and non-uniform compared with the SPgV_krc_ samples described above, precluding an assessment of diversity across each ORF. On the other hand, the single SPgV_kbab_ detected had an average coverage of 402 reads, with 98% ORF coverage of greater than 100 reads, yet this sample exhibited only two SNPs applying the same 5% SNP frequency cutoff, both in NS5B.

### Between-host Patterns of Selection

To assess selection along the polyprotein, we aligned three representative Kibale SPgV consensus sequences (accession no. KF234523, KF234526 and KF234530) and determined average substitution rates at non-synonymous (dN) and synonymous (dS) sites, and their ratio (dN/dS), along a sliding window (100 aa window, 20 aa step; Fig. 4B). Consistent with observed within-host variability for SPgV_krc_, dS among Kibale SPgV consensus sequences was steady across the ORF, while dN showed greater variability, increasing notably within E2, P7, NS2 and NS5A and remaining steadily low throughout NS3. Accordingly, dN/dS ratios were low within NS3, suggesting that on average purifying selection acts to remove deleterious mutations from this coding region. In contrast, dN/dS ratios were >1 within E2, NS2 and NS5A, suggesting that positive selection may act to favor sequence diversification in these genes among the Kibale SPgVs.

## Discussion

Here we report the discovery and characterization of novel pegiviruses in red colobus monkeys, red-tailed guenons and an olive baboon from western Uganda, an area that is a center of biodiversity [77] and a proposed “hotspot” for emerging infectious disease [28], [78]. Our study describes the first pegiviruses detected in Old World monkeys and joins other emerging data demonstrating the impressive diversity of viruses infecting the endangered Kibale red colobus [29], [30], [79]. Overall, the high degree of conservation of genetic features among the Kibale SPgVs, HPgV and SPgV_cpz_ suggests conservation of function among these related viruses. In fact, the distance between the Kibale SPgVs and HPgV is considerably less than the distance between HCV and its closest known relative, NPHV, whose natural host is horses [69], [70].

This study supports the idea that pegivirus infection may be common among Old World and New World primates. To date, distinct SPgVs have been recovered from six New World monkey species: four tamarins (*Saguinus labiatus, S. mystax, S. nigricollis and S. oedipus*), an owl monkey (*Aotus trivirgatus*) and a marmoset (*C. jacchus*) [2], [7], [72]. Infection rates determined in wild-caught New World primates were generally high: 13 of 37 *S. mystax*, 7 of 9 *S. nigricollis*, 3 of 12 *S. labiatus*, 2 of 4 *S. oedipus*, 2 of 2 *C. jacchus*, and 6 of 12 *A. trivirgatus* [7]. Prior to our study, knowledge of pegivirus infections in Old World primates was limited to chimpanzees and humans: SPgV_cpz_ viremia was previously detected in 3 of 39 wild and 6 of 235 captive chimpanzees [8], [35], and the high prevalence of HPgV infection has been widely documented [18]. Here, a large proportion of the red colobus monkeys (28 of 60) and red-tailed guenons (5 of 12) sampled were viremic, suggesting that infections are common. In contrast, a single baboon (of 23 sampled) was positive, precluding similar inferences.

The Kibale SPgVs are monophyletic and share a most-recent common ancestor with HPgV and SPgV_cpz_, demonstrating the closely shared evolutionary history of these viruses in a geographic region where HPgV shows significant genetic variability [75]. Analysis of the partial-helicase phylogeny allows for comparison of the genetic diversity of most primate pegiviruses described to date across a well-studies coding sequence: the clades comprising pegiviruses that infect Old versus New World primate species now exhibit similar genetic diversity, while sequences of Kibale SPgV variants from animals of a given species were highly similar (≥94% nt ID). Considering the much greater within-species diversity of known SPgV_cpz_ variants (73% nt ID) and of several SPgV variants infecting New World monkeys (80 to 90% minimum nt ID within each species), the potential exists for significant undiscovered diversity in SPgVs infecting other populations of primates.

In contrast to the monophyletic sorting of Old World primate pegiviruses, the paraphyletic (i.e., multi-lineage) distribution of hepaci- and pegiviruses infecting bats and rodents is striking [12], [14], [15], [61]. While this pattern may reflect the extensive sampling of these host taxa compared to primates, it also suggests extensive historical cross-species transmission of viruses within each distantly related genus [74]. For example, it is now clear that the New World SPgVs share a more recent common ancestor with a diverse array of bat pegiviruses than with Old World primate pegiviruses [12]. Furthermore, experimental infection of chimpanzees with HPgV [80] and laboratory passaging of New World SPgV isolates through different tamarin species [6] both demonstrate the capacity for extant pegiviruses to infect some closely related hosts.

Our assessments of within-host genetic diversity and between-host selection help to clarify the role of natural selection on the evolution of the Kibale SPgVs. In SPgV_krc_, we observed a relative abundance of low-frequency SNPs, and an even distribution of synonymous substitutions along the polyprotein. These observations suggest a significant and ongoing contribution of random mutations to SPgV within-host synonymous genetic diversity. On the other hand, we observed very low genetic diversity among the consensus SPgV_krc_ sequences recovered from different animals during a 28-month period. These results indicate that purifying selection is likely acting to remove deleterious mutations in replicating viruses. If the evolution of SPgV_krc_ were selectively neutral or strongly impacted by positive selection, we would expect to see greater genetic diversity among consensus sequences over this time period.

Among the different Kibale SPgVs, the dN/dS ratio, which estimates the relative rate of selected versus neutral changes across consensus sequences [81], varied across the polyprotein. We observed dN/dS values exceeding one in E2, NS2, and NS5A, suggesting positive selection on these genes. Interestingly, within individual SPgV samples – SPgV_krc_ in particular – the distribution of nonsynonymous substitutions was similarly biased, with their prevalence in E2, P7, NS2 and NS5A suggesting greater tolerance and/or greater functional significance of nonsynonymous mutations in these genes. Similarly, across NS3, the absence of nonsynonymous substitutions within individual samples combined with low and steady dN/dS ratios for each of the Kibale SPgVs signify the impacts purifying selection on this functionally important and constrained gene [82]. For NS5B we also observed low dN/dS ratios. This pattern is consistent with purifying selection, reflecting the region’s critical role in RNA replication.

Persistent HPgV viremia is associated with prolonged survival and improved surrogate markers of disease progression in HIV-positive individuals [18], [83], [84]. Several potential mechanisms for the apparent protective effects of HPgV viremia have been identified, including direct antiviral effects; altered expression of cytokines, chemokines and HIV entry receptors; and modulation of host cell signaling pathways [18], [19], [27]. However, a great deal of uncertainty still surrounds HPgV mitigation of HIV pathogenesis, in part because no tractable animal model exists with which to study this phenomenon. It is noteworthy that natural SIV infection is present in the primates of western Uganda [79]. Whether SIV and SPgV interact in co-infected hosts is currently unclear. Despite early reports [85], [86], HPgV has not been shown to infect macaques, the most developed and well-understood animal model of HIV pathogenesis, presumably because the barriers to host switching between humans and macaques are too great [3]. We speculate that these barriers might be more easily surmounted by the SPgVs described here, as macaques are much more closely related to the Old World monkey hosts of these viruses than they are to humans [87]. If SPgV variants productively infect macaques, SPgV-SIV coinfections may provide new avenues for understanding the mechanisms by which persistent HPgV infection antagonizes HIV pathogenesis.

## Materials and Methods

### Ethics Statement

The use of animal samples in this study followed the guidelines of the Weatherall Report on the use of non-human primates in research and was approved by the Uganda Wildlife Authority, the Uganda National Council for Science and Technology, and the University of Wisconsin Animal Care and Use Committee prior to initiation of the study.

### Study Site and Sample Collection

This study was conducted in Kibale National Park, western Uganda (0°13′–0°41′ N, 30°19′–30°32′ E), with prior approval from the Uganda National Council for Science and Technology and the Uganda Wildlife Authority. Kibale National Park is a semi-deciduous forest (795 km^2^) located near the Rwenzori Mountains and is notable for its biodiversity and density of primates [28], [77]. Monkeys were immobilized and plasma was sampled as previously described [29]. Blood samples discussed in the current report were collected from January 2010 to June 2012, separated using centrifugation in a field laboratory, and frozen immediately in liquid nitrogen for storage and transport. All animal protocols received prior approval from the Uganda National Council for Science and Technology, the Uganda Wildlife Authority, and the University of Wisconsin Animal Care and Use Committee. All samples were shipped in accordance with international laws under Ugandan CITES permit #002290.

### RNA Extraction and Deep Sequencing

One ml blood plasma from each animal was filtered (0.45 µm) to remove residual host cells, and viral RNA was isolated using the Qiagen QIAamp MinElute virus spin kit (Qiagen, Hilden, Germany), omitting carrier RNA. The eluted RNA was treated with DNase I (DNA-free, Ambion, Austin, TX, USA), and double-stranded DNA was generated using the Superscript double-stranded cDNA Synthesis kit (Invitrogen, Carlsbad, CA, USA), primed with random hexamers. The DNA was purified using the Agencourt Ampure XP system (Beckman Coulter, Brea, CA, USA) and approximately 1 ng DNA was prepared for sequencing on an Illumina MiSeq (Illumina, San Diego, CA, USA) using the Nextera DNA sample preparation kit (Illumina, San Diego, CA, USA). Sequence data were analyzed using CLC Genomics Workbench version 5.5 (CLC bio, Aarhus, Denmark). Briefly, low-quality (CLC quality trimming limit = 0.001; phred quality score <30) and short reads (<100 bp) were removed and the remaining reads were subjected to *de novo* assembly. Assembled contiguous sequences (contigs) were queried against the GenBank database using the basic local alignment search tools blastn and blastx.

### Quantitative RT-PCR

Pegivirus titers were determined via TaqMan quantitative RT-PCR (qRT-PCR). RNA was extracted from 200 µL plasma using the Qiagen QIAamp MinElute virus spin kit (Qiagen, Hilden, Germany). Quantification was performed on a Lightcycler 480 real-time instrument (Roche, USA) using the SuperScript III Platinum One-Step Quantitative RT-PCR System kit (Invitrogen, Grand Island, NY). Pegiviruses detected in plasma from red colobus and red-tailed guenons were quantified using the following oligonucleotides, which were designed to anneal with all known Old World primate pegiviruses, including those described in the present work: forward 5′–TACGACGACTGCCCITACAC–3′ (750 nM); reverse 5′–TTTGCCCAGCTIACATCAGG–3′ (750 nM); probe 5′–FAM–CGCAGCCGTCGCTGCTGACAT–BHQ1–3′ (150 nM). The pegivirus detected in plasma from an olive baboon was quantified using a separate assay, which was optimized to detect this virus: forward 5′–CGGTGTTCATGGCAGGTAT–3′ (500 nM); reverse 5′–AAACACGCGGCTGTAACTG–3′ (500 nM); probe 5′–FAM–ATGCACCCTGATGTAAGCTGGGCAA–BHQ1–3′ (100 nM). Assays were sensitive to between 10 and 100 genome copies per 20-µL qRT-PCR reaction. Standard cycling conditions for the SuperScript III qRT-PCR kit were used, with an annealing temperature of 58°C. Species-specific RNA standard curves were prepared from viral RNA that was (i) amplified with primers flanking each qRT-PCR amplicon (primer sequences are available upon request), (ii) cloned (Zero Blunt PCR Cloning Kit; Invitrogen), (iii) linearized (Hind III digestion; NEB, Ipswich, MA), (iv) transcribed (MEGAscript T7 kit; Ambion, Grand Island, NY), and (v) quantified fluorometrically (Qubit 2.0, Invitrogen, Grand Island, NY).

### Genome Characterization

Mature *Flaviviridae* structural proteins are cleaved from a single polyprotein by host signal peptidase and were distinguished here through manual [46] and *in silico* [47], [48] signalase cleavage site prediction. N-glycosylation of envelope proteins was predicted using N-GlycoSite [88]. RNA secondary structure analysis of 5′- and 3′-untranslated regions (UTRs) was conducted using the RNAfold and RNAalifold algorithms, executed on the Vienna RNA Websuite server [38], [39]. Full and targeted portions of UTR alignments were analyzed to obtain consensus structures of regions with significant structural conservation. The molecular weight of the putative ion channel protein, p7, was estimated using the *p*I/MW tool available on the ExPASy Bioinformatics Resource Portal [49]. Sequence-based analyses of NS5A to identify intrinsically disordered regions (IDRs) and sites within IDRs with capacity to undergo disorder-to-order transitions for binding interactions were conducted with the ANCHOR software package version 1.0 [63], [64]. Linear motifs within IDRs potentially involved in protein-protein or substrate-protein interactions were identified by querying the Eukaryotic Linear Motif (ELM) database, using a conservative motif probability cutoff of 0.01 [65]. Sequences of the pegiviruses described in this manuscript were deposited in the Genbank database under accession numbers KF234499 to KF234530.

### Phylogenetic and Sequence Similarity Analyses

Complete coding sequences of 44 viruses available in GenBank (as of June 2013) were included in the phylogenetic analysis to capture the maximum diversity within known major clades of the hepaci- and pegiviruses. Codon-guided sequence alignments were generated via MAAFT and cleaned using Gblocks [89] using TranslatorX [90]. A Bayesian phylogenetic tree was constructed using MrBayes version 3.2.1 (23). Only the first two codon positions of the nucleotide alignment were considered, since third codon positions demonstrated significant substitution saturation (*p*<0.0001), as determined using DAMBE version 5.3.38 [91]. Characters in the input alignment were partitioned by codon position, and model parameters were estimated independently from the data under default priors. Markov chains were run for 10 million generations, and robustness of phylogenetic groupings was assessed using posterior probability values calculated in MrBayes. The resulting majority rule consensus tree was displayed using FigTree version 1.3.1. The substitution model used in this analysis, GTR+I+Γ, was selected using jModelTest [92], and was based on the 5502-character alignment retained after Gblocks treatment and removal of third-codon-position nucleotides.

We also conducted phylogenetic analyses using a conserved 97-aa segment of the NS3 helicase gene commonly used for the taxonomy of hepaci- and pegiviruses (polyprotein positions 1221 to 1317 with reference to HPgV, NC_001710) [7]–[9], [72], [73]. Following the codon-based alignment of 43 pegivirus and 25 hepacivirus sequences, we generated a neighbor-joining phylogeny (Poisson-corrected p-distance model, pairwise deletion, uniform rates) using MEGA5 (version 5.05) [93], with 5,000 bootstrap replicates of the data to assess the statistical confidence of phylogenetic groupings. Neighbor-joining methods based on amino-acid alignments are commonly used to classify novel hepaci- and pegiviruses when highly conserved genes (e.g., NS3 or NS5A) are targeted [14], [61].

Recombination among Old World primate pegiviruses was assessed using GARD [94] and RDP3 [95]. Sequence identity (p-distance) was determined using MEGA5. Amino acid similarity between the novel and related primate pegiviruses was plotted across codon-aligned genomes by the sliding-window method implemented in SimPlot version 3.5.1 [96]. Because no recombinants were identified (see below), no sequences were excluded from phylogenetic analyses.

### Within-host Genetic Diversity

Single nucleotide polymorphism (SNP) analysis was performed using CLC’s SNP analysis tool as previously described [61]. Stringent variant-calling criteria were used to ensure that only high-quality and high-coverage areas were considered in SNP calling (window = 7; maximum gap and mismatch count = 2; minimum central quality base = 30; minimum average quality for window = 25; minimum coverage = 100×; minimum variant frequency = 5%; SNP required in both forward and reverse reads). At the minimum accepted coverage of 100 high-quality reads and considering the requirement that SNPs be present in the forward and reverse direction, the theoretical detection threshold of this method is two SNPs, or 2%. Therefore, considering variants at a minimum frequency of 5% excludes any singleton observations (i.e., SNPs present on a single library fragment) and provides a conservative estimate of biologically relevant within-host genetic variation. This method also allows for direct comparisons with hepaci- and pegiviruses identified in previous studies [61], [75].

### Between-host Selection

We aligned three representative pegivirus consensus sequences using via MAAFT, one from each positive monkey species (accession no. KF234523, KF234526 and KF234530), and estimated substitution rates at non-synonymous (dN) and synonymous (dS) sites, and their ratio (dN/dS), according to the method described by Nei and Gojobori [97] using the SNAP tool [98]. Values determined by SNAP for each amino acid in the alignment were then average along a sliding window (100 aa window, 20 aa step) for display.

## Supporting Information

Figure S1 **Observed viral titers for the three novel Kibale simian pegiviruses, SPgV_krc_, SPgV_krtg_ and SPgV_kbab_, determined via quantitative TaqMan qRT-PCR.**
(EPS)

Figure S2 **Conserved RNA secondary structure in the 5′- and 3′-untranslated regions (UTRs) of Old World primate pegiviruses.** Targeted portions of UTR alignments were analyzed using RNAalifold to obtain consensus structures and associated base-pair probabilities. Structures were numbered according to schemes established previously for pegivirus 5′- [36] and the 3′-UTRs [44]. Alignment positions along structures are listed for two reference sequences, HPgV strain Iowan (AF121950) and SPgV_krc__RC01 (KF234505), respectively. The alignments analyzed comprised sequences from five red colobus (KF234505, KF234499, KF234523, KF234521, KF234520), one baboon (KF234530), two red-tailed guenon (KF234528, KF234529), one chimpanzee (AF070476) and five human (AF121950, U44402, KC618398, KC618400, KC618401) pegiviruses. Of note, sequence comprising the stem of stem-loop VII was absent in the 3′UTR of SPgV_cpz_.
(EPS)

Figure S3 **The distribution of synonymous substitutions, aggregated from 13 high-coverage SPgV_krc_ samples, was proportional to the gene length across the ORF, suggesting a steady and essentially homogeneous contribution of random mutations to within-host synonymous genetic diversity.**
(EPS)

Figure S4 **The distribution of nonsynonymous substitutions, aggregated from 13 high-coverage SPgV_krc_ samples, was poorly correlated to the gene length across the ORF, suggesting a differential impact of natural selection on within-host nonsynonymous genetic diversity.**
(EPS)

Table S1 **Taxa included in the Bayesian phylogenetic analysis (** **Fig. 2** **).**
(PDF)

Table S2 **Taxa included in the NS3 helicase neighbor-joining phylogenetic analysis (** **Fig. 3** **) in addition to those listed in Table S1. All taxa listed in Table S1 were included in this analysis.**
(PDF)

Table S3 **The distribution of synonymous and nonsynonymous substitutions, aggregated from 13 high-coverage SPgV_krc_ samples, along the polyprotein versus gene length.**
(PDF)
