# Supplementary material for: Discovery and Characterization of Distinct Simian Pegiviruses in Three Wild African Old World Monkey Species
Source: PLoS One. 2014 Jun 11;9(6):e98569. doi: 10.1371/journal.pone.0098569 (PMC4053331; doi:10.1371/journal.pone.0098569)
Supplement: Table S1 — Taxa included in the Bayesian phylogenetic analysis ( Fig. 2 ). (PDF) [file pone.0098569.s005.pdf]

Table S1: Taxa included in the Bayesian phylogenetic analysis (Fig. 2)

Label	Accession no.	Genus	Virus
**SPgVkrtg-K06**	**KF234526**	***Pegivirus***	**Simian pegivirus krtg 06**
**SPgVkbab-K23**	**KF234530**	***Pegivirus***	**Simian pegivirus kbab 23**
**SPgVkrc-K13**	**KF234523**	***Pegivirus***	**Simian pegivirus krc 13**
GHV-1	KC551801	*Hepacivirus*	Guereza hepacivirus BWC05
GHV-2	KC551802	*Hepacivirus*	Guereza hepacivirus BWC04
GBV-B	NC_001655	*Hepacivirus*	Hepatitis GB virus B
HCV-1	NC_004102	*Hepacivirus*	Hepatitis C virus genotype 1
HCV-2	NC_009823	*Hepacivirus*	Hepatitis C virus genotype 2
HCV-3	NC_009824	*Hepacivirus*	Hepatitis C virus genotype 3
HCV-4	NC_009825	*Hepacivirus*	Hepatitis C virus genotype 4
HCV-5	NC_009826	*Hepacivirus*	Hepatitis C virus genotype 5
HCV-6	NC_009827	*Hepacivirus*	Hepatitis C virus genotype 6
HCV-7	EF108306	*Hepacivirus*	Hepatitis C virus genotype 7
NPHV	JF744991	*Hepacivirus*	Non-primate hepacivirus
NPHV	JQ434002	*Hepacivirus*	Non-primate hepacivirus
RHV	NC_021153	*Hepacivirus*	Rodent hepacivirus 339
BHV-829	KC796074	*Hepacivirus*	Bat hepacivirus 829
BHV-112	KC796077	*Hepacivirus*	Bat hepacivirus 112
BHV-452	KC796090	*Hepacivirus*	Bat hepacivirus 452
HPgV	NC_001710	*Pegivirus*	Human pegivirus
HPgV	AB003291	*Pegivirus*	Human pegivirus
HPgV	AB013500	*Pegivirus*	Human pegivirus
HPgV	D87708	*Pegivirus*	Human pegivirus
HPgV	HQ331233	*Pegivirus*	Human pegivirus
HPgV	KC618398	*Pegivirus*	Human pegivirus
SPgV-A	U22303	*Pegivirus*	Simian pegivirus A
SPgVcal-mx	AF023424	*Pegivirus*	Simian pegivirus cal-mx
SPgVlab	NC_001837	*Pegivirus*	Simian pegivirus lab-T1059
SPgVtri	AF023425	*Pegivirus*	Simian pegivirus tri-1122
SPgVcpz	AF070476	*Pegivirus*	Simian pegivirus cpz
BPgV	GU566735	*Pegivirus*	GB virus D
BPgV-24	KC796082	*Pegivirus*	Bat pegivirus 24
BPgV-34.1	KC796093	*Pegivirus*	Bat pegivirus 34.1
BPgV-76.1	KC796084	*Pegivirus*	Bat pegivirus 76.1
BPgV-303	KC796073	*Pegivirus*	Bat pegivirus 303
BPgV-491.2	KC796089	*Pegivirus*	Bat pegivirus 491.2
BPgV-694	KC796083	*Pegivirus*	Bat pegivirus 694
BPgV-737B	KC796081	*Pegivirus*	Bat pegivirus 737B
BPgV-838	KC796086	*Pegivirus*	Bat pegivirus 838
BPgV-1715	KC796088	*Pegivirus*	Bat pegivirus 1715
BPgV-1734	KC796087	*Pegivirus*	Bat pegivirus 1734
EqPgV	NC_020902	*Pegivirus*	Equine pegivirus
EqPgV-TDAV	KC145265	*Pegivirus*	Equine pegivirus TDAV
RPgV-CC61	KC815311	*Pegivirus*	Rodent pegivirus CC61
