# Supplementary material for: Discovery and Characterization of Distinct Simian Pegiviruses in Three Wild African Old World Monkey Species
Source: PLoS One. 2014 Jun 11;9(6):e98569. doi: 10.1371/journal.pone.0098569 (PMC4053331; doi:10.1371/journal.pone.0098569)
Supplement: Table S2 — Taxa included in the NS3 helicase neighbor-joining phylogenetic analysis ( Fig. 3 ) in addition to those listed in Table S1. All taxa listed in Table S1 were included in this analysis. (PDF) [file pone.0098569.s006.pdf]

Table S2: Taxa included in the NS3 helicase neighbor-joining phylogenetic analysis (Fig. 3) in addition to those listed in Table S1. All taxa listed in Table S1 were included in this analysis.

Label	Accession no.	Genus	Virus
**SPgVkrtg-K11**	**KF234529**	***Pegivirus***	**Simian pegivirus krtg 11**
**SPgVkbab-K23**	**KF234530**	***Pegivirus***	**Simian pegivirus kbab 23**
**SPgVkrc-K54**	**KF234520**	***Pegivirus***	**Simian pegivirus krc 54**
RHV-pm4144	KC815326	*Hepacivirus*	Rodent hepacivirus pm4144
RHV-pm4062	KC815324	*Hepacivirus*	Rodent hepacivirus pm4062
RHV-pm3252	KC815322	*Hepacivirus*	Rodent hepacivirus pm3252
RHV-pm3243	KC815320	*Hepacivirus*	Rodent hepacivirus pm3243
RHV-ch2110	KC815318	*Hepacivirus*	Rodent hepacivirus ch2110
RHV-pm3038	KC815327	*Hepacivirus*	Rodent hepacivirus pm3038
RHV-pm4109	KC815325	*Hepacivirus*	Rodent hepacivirus pm4109
RHV-nl4106	KC815323	*Hepacivirus*	Rodent hepacivirus nl4106
RHV-pm5198	KC815321	*Hepacivirus*	Rodent hepacivirus pm5198
RHV-pm5263	KC815319	*Hepacivirus*	Rodent hepacivirus pm5263
RHV-089	KC815312	*Hepacivirus*	Rodent hepacivirus 089
RPgV-pm5226	KC815317	*Pegivirus*	Rodent pegivirus pm5226
RPgV-pm6041	KC815316	*Pegivirus*	Rodent pegivirus pm6041
RPgV-pm6073	KC815315	*Pegivirus*	Rodent pegivirus pm6073
RPgV-pm6087	KC815313	*Pegivirus*	Rodent pegivirus pm6087
RPgV-pm6197	KC815314	*Pegivirus*	Rodent pegivirus pm6197
SPgVlab-119	U84947	*Pegivirus*	Simian pegivirus lab 119
SPgVlab-140	U84948	*Pegivirus*	Simian pegivirus lab 140
SPgVmys-156	U84949	*Pegivirus*	Simian pegivirus mys 156
SPgVmys-750	U84952	*Pegivirus*	Simian pegivirus mys 750
SPgVmys-760	U84953	*Pegivirus*	Simian pegivirus mys 760
SPgVmys-795	U84956	*Pegivirus*	Simian pegivirus mys 795
SPgVnig-17	U84961	*Pegivirus*	Simian pegivirus nig 17
SPgVnig-18	U84962	*Pegivirus*	Simian pegivirus nig 18
SPgVnig-95	U84963	*Pegivirus*	Simian pegivirus nig 95
SPgVoed-100	U84965	*Pegivirus*	Simian pegivirus oed 100
SPgVoed-14	U84966	*Pegivirus*	Simian pegivirus oed 14
SPgVtri-924	U84944	*Pegivirus*	Simian pegivirus tri 924
SPgVtri-933	U84946	*Pegivirus*	Simian pegivirus tri 933
SPgVcpz-33	AF068913	*Pegivirus*	Simian pegivirus cpz 33
