# Supplementary material for: Discovery and Characterization of Distinct Simian Pegiviruses in Three Wild African Old World Monkey Species
Source: PLoS One. 2014 Jun 11;9(6):e98569. doi: 10.1371/journal.pone.0098569 (PMC4053331; doi:10.1371/journal.pone.0098569)
Supplement: Table S3 — The distribution of synonymous and nonsynonymous substitutions, aggregated from 13 high-coverage SPgVkrc samples, along the polyprotein versus gene length. (PDF) [file pone.0098569.s007.pdf]

Table S3: The distribution of synonymous and nonsynonymous substitutions, aggregated from 13 high-coverage SPgVkrc samples, along the polyprotein versus gene length.

	**Gene Length**		**Synonymous**		**Nonsynonymous**	
**Gene**	nt	% of complete ORF	No.	% of total no.	No.	% of total no.
N.A.	45	0.5	0	0	0	0
E1	570	7	21	7	3	6
E2	1122	13	50	16	14	29
P7	204	2	7	2	6	12
NS2	714	8	29	9	10	20
NS3	1881	22	70	22	0	0
NS4A	183	2	5	2	0	0
NS4B	840	10	21	7	0	0
NS5A	1443	17	55	17	8	16
NS5B	1692	19	60	19	8	16
